# Gut microbiota dysbiosis and immune responses: insights from IgA nephropathy and inflammatory bowel disease

**DOI:** 10.3389/fimmu.2026.1889646

**Published:** 2026-07-29

**Authors:** Lu Yi, Ran Zhang, Yang Yang, Xiaoli Wen, Yu Wang, Fang Zeng, Gaosi Xu

**Affiliations:** 1 Department of Nephrology, The Second Affiliated Hospital, Jiangxi Medical College, Nanchang University, Nanchang, China; 2 Department of Nephrology, Ganzhou Hospital-Nanfang Hospital, Southern Medical University (Ganzhou People’s Hospital), Ganzhou, China

**Keywords:** gut microbiota, gut-kidney axis, IgA nephropathy, inflammatory bowel disease, mucosal immunity

## Abstract

IgA nephropathy (IgAN) is the most common glomerulonephritis worldwide, and inflammatory bowel disease (IBD) comprises a group of chronic, relapsing inflammatory bowel disorders. Although IgAN and IBD differ in clinical manifestations, both involve interactions among genetic susceptibility, epigenetic abnormalities, and gut microbiota dysbiosis, ultimately pointing to mucosal immune dysregulation and chronic inflammation. They share similar alterations in gut microecology, characterized by a reduction in short-chain fatty acid-producing protective commensals and an increase in pathogenic symbionts. This leads to intestinal barrier disruption and aberrant microbial metabolites, promoting the production of galactose-deficient IgA1 (Gd-IgA1), activating intestinal mucosal immune cells, and driving both local and systemic inflammation. Notably, glucocorticoids (GC) and immunomodulators are common therapeutic agents for both diseases, and effective treatment is accompanied by restoration of the gut microbiota and improvement of immune imbalance. The four currently marketed budesonide enteric-coated capsules employ pH-dependent targeted-release technology and act on different sites ranging from the terminal ileum to the colon to modulate B-cell activation and attenuate local inflammation. IgAN and IBD may be closely related mucosal immune disorders, with immune inflammation associated with gut microbiota dysbiosis may represent a key link between them. Furthermore, upper respiratory tract infection may participate in the development of IgAN through mucosal immunity. The efficacy of tonsillectomy as a therapeutic intervention suggests the existence of crosstalk between the respiratory tract and the kidneys.

## 1 Introduction

IgA nephropathy (IgAN) is the most common primary glomerulonephritis worldwide, characterized by predominant IgA deposition in the glomerular mesangium (1). The disease can occur at any age but predominantly affects young adults. Typical clinical presentation includes macroscopic hematuria following respiratory or gastrointestinal infections, often accompanied by varying degrees of proteinuria, hypertension, or renal impairment, and may eventually progress to end-stage renal disease (ESRD) (2). The incidence of IgAN varies geographically, being most common in East Asia and less frequent in South America and Africa, with genetic risk and environmental exposures playing important roles (3). The “four-hit theory” is currently a classic model. Recently, the concept of a “five-hit theory” has been proposed, prompting reconsideration of the initial trigger of IgAN. Professor Trimarchi suggests that polygenic regulation precedes the classic four hits, defining this as “hit 0.” This phase involves interactions among genetic, epigenetic, and microbiome factors, determining why an individual overproduces aberrantly glycosylated IgA1. Additionally, the concept of the gut-kidney axis has deepened our understanding of IgAN pathogenesis. The gut microbiota and the kidneys interact bidirectionally through microbial metabolites. Gut dysbiosis has been associated with compromise of the intestinal barrier, which may facilitate the translocation of bacteria and toxins into the circulation and contribute to kidney injury. Conversely, kidney failure leads to the accumulation of uremic toxins, which further disrupt the gut microecology, establishing a vicious cycle (4). Mucosal immunity plays a crucial role in IgAN progression, primarily via the gut–kidney axis. Under the influence of internal and external factors such as genetics and environment, alterations in the gut-associated mucosal immune system promote disease progression (5). This aligns with the current four-hit theory, which maintains that galactose-deficient IgA1 (Gd-IgA1) originates mainly from the gut-associated lymphoid tissue (GALT) (6). Inflammatory bowel disease (IBD) is a chronic inflammatory condition of the digestive tract, typically encompassing Crohn’s disease (CD) and ulcerative colitis (UC). The pathogenesis of IBD is multifactorial, involving a complex interplay among genetic susceptibility, environmental triggers, gut microbiota dysbiosis, and mucosal immune dysregulation (7).

Genome-wide association studies (GWAS) have identified shared susceptibility genes between IgAN and IBD, and these genes are involved in immune regulation and mucosal barrier function. Several case reports have described the coexistence of the two diseases, with IBD being diagnosed before IgAN in most instances, suggesting that patients with IBD have a higher risk of developing IgAN (8–10). Clinical cases also indicate that relapses and remissions of IgAN often parallel IBD disease activity. Both conditions feature intestinal mucosal damage and dysbiosis, and drugs targeting gut mucosal immunity, such as topical glucocorticoids (GC), are effective in both IgAN and IBD, further suggesting the presence of shared, actionable therapeutic targets.

In this review, we aim to elucidate the immune dysregulation underlying IgAN development from the perspective of the “five-hit” theory, explore the intrinsic link between IBD and IgAN comorbidity from a gastroenterological viewpoint, and examine the interplay between gut microbiota dysbiosis and systemic inflammation in these two diseases. We attempt to propose a unifying hypothesis: both IgAN and IBD develop on the basis of genetic and epigenetic susceptibility, and are triggered by local factors from the gut and/or respiratory mucosa, which initiate systemic inflammatory immune responses. In turn, effective control of local and circulating inflammation and immune responses may restore the mucosal microenvironment and break this vicious cycle.

To identify relevant literature, we primarily searched the PubMed database covering the period from database inception to May 2026, using keywords including “IgA nephropathy”, “inflammatory bowel disease”, “gut microbiota”, and “mucosal immunity” in various combinations. Additionally, we manually screened the reference lists of the retrieved articles to supplement additional important studies. The final selection of literature was based on our comprehensive assessment of topical relevance and scholarly quality. Given the narrative nature of this review, we did not perform a systematic quantitative synthesis or apply formal inclusion/exclusion criteria.

## 2 Genetic background of IgAN and IBD

### 2.1 Shared comorbidity genes revealed by GWAS

Extensive research has identified genetic susceptibility loci for IgAN. Kiryluk et al. studied 20,612 individuals of European and East Asian ancestry and identified multiple risk loci for primary IgAN. Several susceptibility genes are shared between IgAN and IBD, primarily affecting pathways related to intestinal mucosal immune defense, antigen presentation, and lymphocyte differentiation, providing genetic evidence supporting the “gut–kidney axis” hypothesis (**Table 1**) (11). Notably, some genetic variants influence disease risk indirectly by modulating the composition of the gut microbiota, suggesting a complex interplay among genetics, microbiota, and immunity.

**Table 1:** Summary of genetic background, epigenetics, gut microbiota changes before and after treatment, and therapeutic aspects in IgA nephropathy, Crohn’s disease, and ulcerative colitis.

Characteristic		IgAN	IBD		Ref
			CD	UC	
Genetic background		HLA-DQ/DR, CARD9, HORMAD2, ITGAM-ITGAX, VAV3, DEFA,TNFSF13, CFHR1, CFHR3	NOD2, ATG16L1, IRGM TNFSF15, LRRK2, IL23R, CARD9, HORMAD2, HLA-DRB1, RIPK2	IL23R, TNFSF15, IL10, HERC2, ECM1, STAT3, PTPN2, ARPC2, CARD9, HORMAD2, HLA-DRB1	(11, 20, 27–30, 101)
Epigenetics		Upregulation of miR-148b inhibits C1GALT1, Upregulation of let-7b inhibits CALNT2, High methylation of the COSMC promoter leads to downregulation of its partner protein and reduced C1GALT1 activity	DEFA5/TNF methylation (CD), reduced methylation of the KHDC3L gene (UC), Low methylation of RPS6KA2, miR-223 suppresses NLRP3 to alleviate intestinal inflammation		(33–35, 37, 38, 101, 102)
Gut microbiota	Before treatment	↑: Streptococcus, Paraprevotella, Escherichia-Shigella, Bacteroides, Pseudomonas, Defluviitaleaceae_incertae_sedis ↓: Fusicatenibacter, Clostridium, Prevotella 9, Roseburia, Barnesiella, Bifidobacterium, members of Ruminococcaceae and Lachnospiraceae families, Parasutterella, Dialister, Faecalibacterium, Subdoligranulum	↑: Proteobacteria, Enterobacteriaceae, Streptococcaceae, Enterococcus, Pyramidobacter, Prevotella, Ruminococcus gnavus group, Haemophilus, ↓: Firmicute, Lachnospiraceae, Faecalibacterium, Roseburia, unclassified Lachnospiraceae, Megamonas, Blautia, Dialister, Phascolarctobacterium	↑: Proteobacteria, Actinomyces, Klebsiella, Limosilactobacillus, Streptococcus, Escherichia-Shigella ↓: Bacteroides, Parabacteroides, Prevotella, Faecalibacterium	(40, 49, 50, 52, 56–58, 74, 78, 103–105)
	After treatment	↑: Clostridiaceae_Clostridium sensu stricto, genus Prevotella ↓: Escherichia-Shigella, Phylum Proteobacteria, Oxalobacter, Butyricoccus	↑: Subdoligranulum, Lachnospira, Clostridiales, Agathobacter, Proteobacteria, Veillonellaceae ↓: Escherichia-Shigella, Bacteroidota, Bacteroidia, Bacteroidales	↑: Bacteroidetes, Faecalibacterium prausnitzii, Bifidobacterium, Akkermansia muciniphila, Lactobacillus, Clostridium, Roseburia ↓: Proteobacteria, Staphylococcaceae, Comamonadaceae, Corynebacteriaceae	
Treatment		RAS inhibitors, SGLT2 inhibitors, DEARA, MRA, ERA, Nefecon (budesonide-targeted), Systemic glucocorticoids, Factor B inhibition, Anticomplement	Topical glucocorticoids, Systemic glucocorticoids, Anti-TNF (infliximab/adalimumab), Anti-IL-12/23 (ustekinumab), JAK inhibitors (upadacitinib), anti-integrin ((vedolizumab), Immunomodulators (azathioprine/methotrexate)	5-ASA, Budesonide MMX, Systemic glucocorticoids, Anti-TNF agents, JAK inhibitors, S1P receptor modulators (ozanimod, etrasimod), Anti-integrin agents, Anti-IL-12/23 agents	(71, 77, 106)

#### 2.1.1 CARD9

In 2014, Kiryluk et al. first reported a significant association between the rs4077515 single nucleotide polymorphism in the *CARD9* gene and IgAN in a large-scale GWAS (11). Notably, this locus has also been confirmed as a risk locus for both CD and UC (12). The *CARD9* gene encodes an adaptor protein containing a CARD (caspase recruitment domain family member) domain, which is a core molecule in innate immune signaling pathways. Through its CARD domain, CARD9 forms a signaling complex with BCL10 and MALT1, activating the NF-κB pathway and promoting the production of pro-inflammatory cytokines such as TNF, IL-6, and IL-1β (13). In IgAN, *CARD9* risk variants are involved in the differentiation of T cells into Th2, Tfh, and Th17 subsets (14), and these T cell subsets play crucial helper roles in IgA class switching in B cells; therefore, *CARD9* variation may promote the production of aberrantly glycosylated IgA1. Furthermore, a study by He et al. showed that IgAN patients carrying the *CARD9* risk allele experience a faster decline in renal function and a higher risk of progressing to ESRD (15). CARD9 signaling is essential for maintaining gut mucosal immune homeostasis; its deficiency leads to reduced IL-22 production and downregulation of antimicrobial peptide expression (16, 17). In IBD, *CARD9* regulates intestinal inflammation via the microbiota-tryptophan metabolism-aryl hydrocarbon receptor (AhR)-IL-22 axis. *CARD9*-knockout mice fail to properly metabolize tryptophan into AhR ligands, resulting in decreased IL-22 and aggravated colitis. IBD patients carrying the *CARD9* risk allele show significantly reduced fecal levels of AhR ligands, confirming that this mechanism also operates in human disease (18).

#### 2.1.2 HLA-DQ/DR

In 2010, Feehally et al. conducted a genome-wide association study of IgAN in a European population and identified a very strong association signal within the *HLA* region, far exceeding signals elsewhere in the genome, with the strongest signal located at the *HLA-DQ* locus (19). In IBD, classical *HLA* genes, particularly class II *HLA-DRB1* and *HLA-DQB1*, are the most strongly associated and most extensively studied risk loci, with *HLA-DRB1* conferring the highest disease risk (20). Class II *HLA* genes are involved in antigen presentation. These loci may contribute to dysregulated intestinal IgA production and the generation of anti-IgA1 IgG antibodies, further exacerbating immune dysregulation in IgAN. Through transcriptomic and genotypic analyses of monocyte-derived macrophages from IBD families, researchers found extreme interindividual variation in the expression levels of *HLA* genes in macrophages. This may affect the efficiency of presenting specific antigenic peptides, subsequently alter T-cell activation and recognition of gut-derived antigens, leading to aberrant immune responses against commensal bacteria or pathogens (21).

#### 2.1.3 HORMAD2

*HORMAD2*, located on chromosome 22q12, is another genetic susceptibility locus shared between IgAN and IBD. A GWAS study reported a significant association between *HORMAD2* and IgAN. Additionally, a significant interaction between *CFHR3/R1* and *HORMAD2* was detected, suggesting that the effect of *HORMAD2* may be modulated by other loci (22). Subsequent studies further confirmed a dose-dependent relationship between the *HORMAD2* risk allele and elevated serum IgA levels; individuals carrying two risk alleles had significantly higher serum IgA concentrations than non-carriers (11). The intronic variant rs2412971 in *HORMAD2* is not only significantly associated with tonsillectomy but also increases the risk of IgAN. This variant may enhance the immune response of mucosa-associated lymphoid tissue to pathogens, promoting the production of pathogenic IgA and thereby participating in IgAN pathogenesis (23). Interestingly, the direction of effect of the *HORMAD2* locus in IBD is opposite to that in IgAN. Serum IgA levels are positively correlated with renal disease and type 2 diabetes, but negatively correlated with IBD and several infections, which may be attributable to the protective anti-inflammatory effect of mucosal IgA (24). This seemingly paradoxical observation does not negate its status as a shared susceptibility locus. One possible interpretation is that HORMAD2 may participate in a common mucosal immune pathway that, in distinct tissue microenvironments, gives rise to disease-specific downstream effects. However, this remains speculative and requires further functional validation.

### 2.2 Disease-specific genetic loci

**Figure 1:**
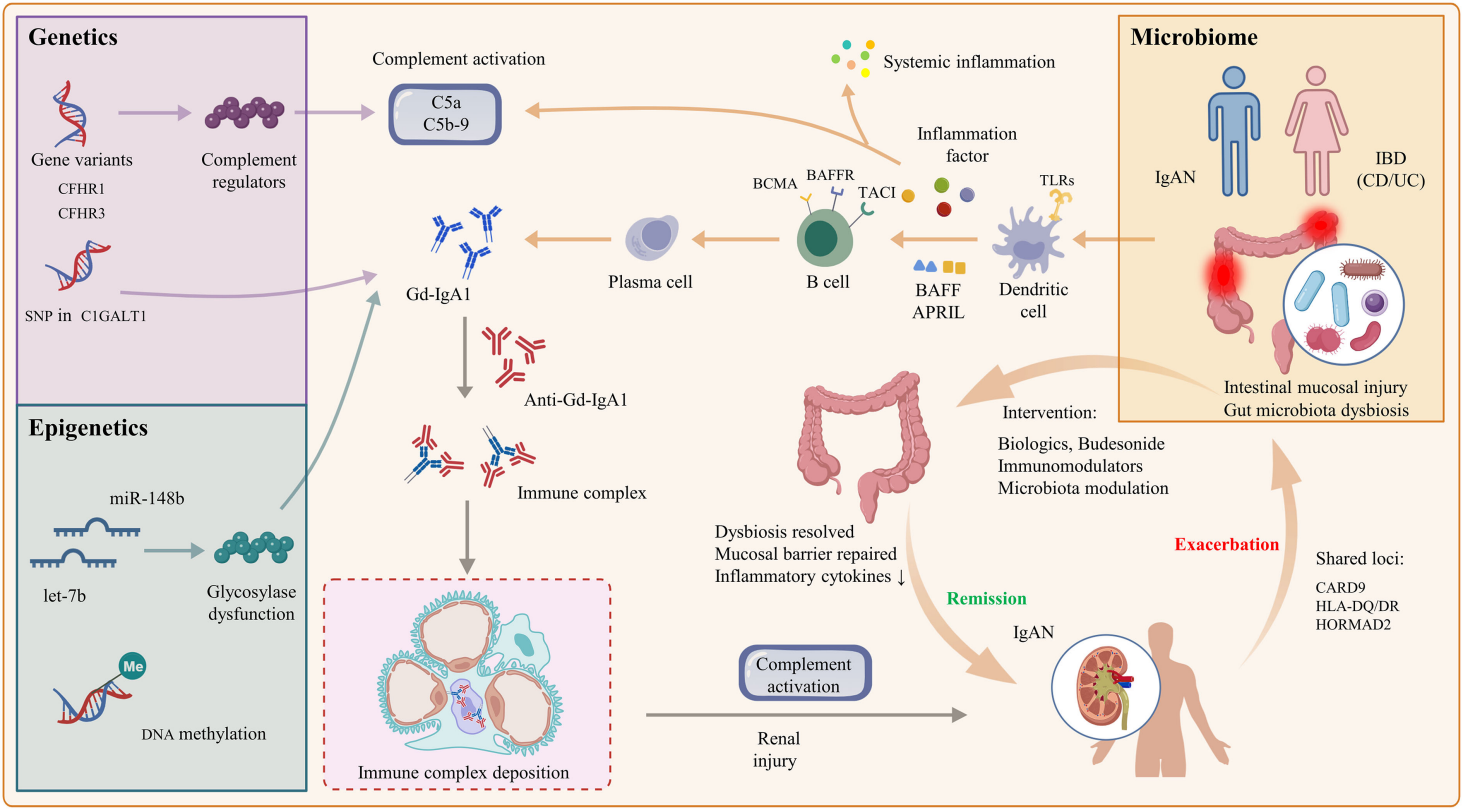
Pathogenesis of IgA nephropathy. Genetic susceptibility, epigenetic regulation, and microbiome interactions collectively constitute the predisposing “Hit 0” phase of the “five-hit theory” in IgA nephropathy (IgAN). Both patients with IgAN and those with inflammatory bowel disease (IBD) exhibit intestinal mucosal damage and dysbiosis, which activate mucosal immunity. Dendritic cells (DCs) activate B cells via Toll-like receptors (TLRs), promoting plasma cell differentiation and the production of aberrantly glycosylated IgA1 (Gd-IgA1). A single nucleotide polymorphism in the non-coding region of *C1GALT1*, a key enzyme involved in O-glycosylation, increases Gd-IgA1 expression in the general population. MicroRNAs such as miR-148b and let-7b regulate the expression of key genes involved in IgA1 O-glycosylation, leading to glycosylation abnormalities. Promoter methylation of certain genes may also contribute, collectively resulting in elevated circulating Gd-IgA1 levels. Gd-IgA1 is recognized by antibodies specific to its hinge region, forming immune complexes that deposit in the glomerular mesangium, thereby inducing mesangial cell activation, proliferation, and complement activation. Additionally, proteins encoded by *CFHR3* and *CFHR1* antagonize complement factor H (CFH), leading to aberrant activation of the alternative complement pathway and subsequent kidney injury. Shared susceptibility genes between IgAN and IBD may contribute to intestinal barrier defects and impaired microbial recognition, thereby exacerbating disease manifestations. Following therapeutic intervention, restoration of the intestinal mucosal barrier and amelioration of dysbiosis are accompanied by clinical improvement in patients with IgAN. Created with BioGDP.com (100).

GWAS studies have identified numerous susceptibility loci for both IgAN and IBD, with partial overlap but also significant disease specificity (**Table 1**). Complement activation is involved in IgAN pathogenesis, and copy number variations of the *CFHR3* and *CFHR1* are significantly associated with IgAN. These genes encode key regulatory proteins of the alternative complement pathway; their genetic variation can affect complement activation, leading to aberrant activation of the alternative pathway that mediates kidney injury (**Figure 1**) (25). This evidence explains why drugs that inhibit complement activation, such as iptacopan, have emerged as a novel targeted therapeutic strategy for IgAN. However, a retrospective single-center cohort of 639 IgAN patients confirmed that the combined deletion of *CFHR3* and *CFHR1* primarily affects complement activation and the level of glomerular immune deposition, but does not influence long-term patient prognosis (26). This finding supports a role for these variants in disease initiation, whereas their involvement in disease progression remains speculative and requires further investigation through longitudinal studies.

*NOD2* is the earliest and strongest genetic susceptibility locus for CD; loss-of-function variants significantly increase CD risk (27–29). It encodes an intracellular pattern recognition receptor that senses bacterial peptidoglycan to initiate antimicrobial immunity (30). *NOD2* deficiency promotes memory CD4^+^ T cell expansion into Th17 cells, with overproduction of IL-17, IL-22, and other pro-inflammatory mediators, thereby exacerbating intestinal inflammation (31). Additionally, *NOD2* negatively regulates toll-like receptor (TLR) -mediated inflammatory responses; its activation induces interferon regulatory factor 4 expression to suppress NF-κB-dependent cytokines and upregulates deubiquitinase A while downregulating TLR9-mediated type I interferon responses. *NOD2* deficiency relieves this inhibition, leading to TLR hyperactivation and chronic intestinal inflammation (32).

## 3 Epigenetic regulation in IgAN and IBD

Currently, IgAN risk loci identified by GWAS explain only part of the disease heritability, suggesting that epigenetic regulation may play an important role in disease development. However, no direct evidence currently exists for shared epigenetic pathways between IgAN and IBD. This section aims to show that epigenetic regulation contributes independently to each disease, yet common patterns persist, as both involve epigenetic dysregulation of key immune−related genes in the mucosal microenvironment.

Aberrant O-glycosylation of the IgA1 hinge region is a key event in IgAN pathogenesis. Polypeptide N-acetylgalactosaminyltransferase 2 (GALNT2) adds N-acetylgalactosamine to the hinge, followed by core 1 β-1,3-galactosyltransferase (C1GALT1)-catalyzed galactose linkage to form Core 1 O-glycan (1, 6). Altered expression of these key glycosyltransferases may contribute to disease. High-throughput miRNA profiling revealed that miR-148b is significantly upregulated in peripheral blood mononuclear cells (PBMCs) of IgAN patients, where it targets the 3’-untranslated region of C1GALT1 and inhibits its expression, which may contribute to increased Gd-IgA1 production (33). The same group further showed that let-7b is also upregulated in PBMCs and B cells of IgAN patients, regulating GALNT2 at mRNA and protein levels (**Figure 1**) (34). In addition, DNA methylation also contributes to aberrant glycosylation in IgAN. Under IL-4 stimulation, hypermethylation of the *COSMC* gene promoter in B lymphocytes from pediatric IgAN patients reduces core 1,3 galactosyltransferase-specific molecular chaperone (COSMC) expression and elevates Gd-IgA1, which can be partially reversed by demethylating agents (35).

Beyond the gut, the tonsils as core upper respiratory mucosal immunity, also contribute to IgAN. Qin et al. found that Cosmc mRNA in peripheral B cells of IgAN patients was reduced but nearly normalized after plasma-free culture; Lipopolysaccharide (LPS) inhibited this recovery (36). Thus, low *COSMC* may arise from external suppression by soluble plasma factors. LPS downregulates *COSMC*, reducing C1GALT1 activity and promoting Gd−IgA1 production.

Altered DNA methylation may serve as a marker of IBD activity. The *RPS6KA2* is hypomethylated in IBD patients, correlating with C-reactive protein and albumin levels, as well as with severe CD behavior and extensive UC lesions (37). miRNAs are also involved in IBD inflammation. For example, myeloid-derived miR-223 is upregulated in IBD patients and colitis mice, limiting inflammation by inhibiting NLRP3 inflammasome. miR-223 deficiency leads to NLRP3 hyperactivation, increased IL-1β, and exacerbated colitis (38).

## 4 Gut microbiota and mucosal immunity: from composition to metabolism

### 4.1 Gut microbiota dysbiosis and immune dysregulation in IgAN and IBD

Genetic and epigenetic studies have linked both IgAN and IBD to common pathways involving gut mucosal immunity. As a key regulator of mucosal immunity, the gut microbiota differs in composition from that of healthy individuals in both diseases (39–42). The gut harbors the highest density of microbes in the human body, reaching 10^11–^10^12^ organisms per gram of tissue (43). Because pH varies markedly along the gastrointestinal tract, distinct bacterial communities colonize different regions. We therefore summarized in **Table 2** the pH ranges and common colonizing bacteria from the stomach to the distal colon based on the literature (44–47).

**Table 2:** Human gastrointestinal pH values and corresponding microbial taxa.

Gastrointestinal site	pH	Predominant microbial families
Stomach	1.5-3.5	Proteobacteria, Firmicutes, Actinomycetota, Bacteroidota, Fusobacteriota, Lactobacillus, Streptococcus, Prevotella
Duodenum	5.5–6.5	Firmicutes, Proteobacteria, Prevotella, Streptococcus, Bacillus, Pseudomonas
Jejunum and ileum	6.0–8.0	Jejunum: Firmicutes, Proteobacteria, Streptococcus, Prevotella, Veillonella, Escherichia Proximal ileum: Micrococcaceae, Streptococcus, Haemophilus, Escherichia Terminal ileum: Enterobacteriaceae, Cetobacterium, Cupriavidus, Bacteroides, Escherichia
Proximal colon	5.0–7.5	Firmicutes, Bacteroidetes, Proteobacteria, Verrucomicrobia, Fusobacteria, Desulfobacterota, Actinobacteria, Faecalibacterium, Escherichia
Distal colon	6.0–8.0	

**Figure 2:**
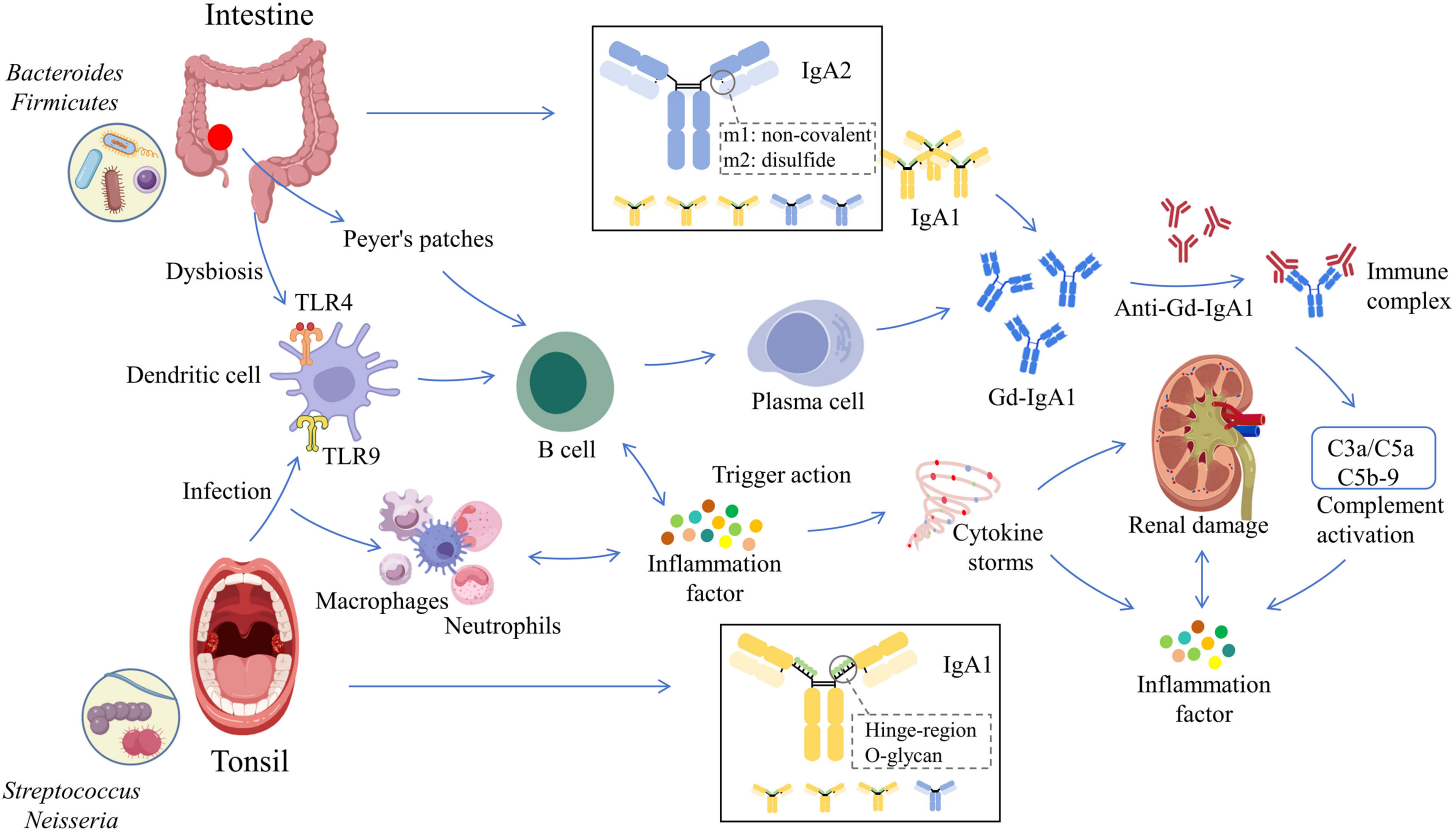
Possible pathogenesis of IgA nephropathy involving the gut and tonsils. Under healthy conditions, the distal ileum and distal intestinal mucosa secrete an increased proportion of IgA2, which has a shorter hinge region and lacks O−glycosylation. One subtype, IgA2 (m2), has its heavy and light chains covalently linked by disulfide bonds, resulting in a stable structure that resists bacterial proteases in the intestine. The predominant commensal bacteria in the gut are Bacteroides and Firmicutes, whereas the tonsillar mucosa is dominated by IgA1 and harbors common bacteria such as Streptococcus and Neisseria. When dysbiosis impairs the intestinal barrier, bacterial products activate submucosal DCs via TLR4. DCs release inflammatory cytokines, which activate B cells. The activated B cells massively differentiate into plasma cells in Peyer’s patches and produce Gd−IgA1, leading to kidney injury through the classic “four−hit” mechanism. The deposited immune complexes activate complement and release inflammatory factors that directly damage the glomerulus. Similarly, upper respiratory tract infections may trigger a comparable B−cell response in the tonsils through DC activation. Meanwhile, recruited innate immune cells release inflammatory factors that can exert a “trigger action,” initiating an inflammatory storm that exacerbates kidney injury. Some of the icons used in this figure were obtained from BioGDP.com (100).

Under healthy conditions, the distal ileum and distal intestinal mucosa secrete an increased proportion of IgA2 (48). IgA2 can be further divided into two subclasses, IgA2 (m1) and IgA2 (m2); the latter has a stable structure with its heavy and light chains covalently linked by disulfide bonds, making it more resistant to intestinal bacterial proteases (**Figure 2**). Gut microbiota dysbiosis can disrupt mucosal immune balance, leading to abnormal IgA production (**Figure 1**) (4). In IgAN, multiple observational studies have consistently observed similar alterations: the opportunistic pathogen *Escherichia-Shigella* increases significantly, while beneficial butyrate-producing bacteria such as *Prevotella* and *Faecalibacterium* decrease (40, 49–51). A large observational study in China not only confirmed that IgAN patients are characterized by a marked increase in *Escherichia-Shigella* but also showed that its abundance positively correlates with more severe clinical and pathological features, consistent with previous reports (52, 53). *Escherichia-Shigella* overgrowth activates the TLR4/MyD88/NF-κB pathway in intestinal B cells via LPS in experimental models, potentially contributing to B-cell overactivation and Gd-IgA1 production (54). Moreover, *Escherichia-Shigella* enrichment has been associated with gut metabolic disturbances and elevates serum sphingosine-1-phosphate (S1P) levels; S1P activates receptor modulation and chemokine ligand 2 (CCL2)-mesenchymal to epithelial transition factor (MET)-focal adhesion kinase (FAK), inducing mesangial proliferation and increasing IgA1 deposition (55). Concurrently, butyrate-producing bacteria are markedly reduced. Compared with healthy controls, IgAN patients show significantly lower abundance of butyrate-producing bacteria. The sulfoquinovose degradation I pathway associated with these bacteria is downregulated, whereas pathways related to immune activation and inflammation are upregulated. Gd-IgA1 levels correlate significantly with these metabolic pathway changes (39). This suggests that reduced butyrate producers may weaken the intestinal barrier and immune regulation, contributing to IgAN onset and progression. These findings indicate that gut-derived IgA may be an important source of pathogenic IgA1 in the circulation.

Despite similarities with IgAN, IBD also exhibits significant gut microbiota dysbiosis. However, while in IgAN dysbiosis acts through increased deposition of abnormal IgA in the kidneys, in IBD it directly exposes the immune system to microbial antigens, driving local inflammation. The gut microbiota in IBD is characterized by reduced α and β diversity, more pronounced in CD (56). Common features include an increase in opportunistic pathogens such as *Escherichia coli* and *Escherichia-Shigella*, and a decrease in butyrate-producing protective bacteria like *Firmicutes* and *Faecalibacterium (*56–58). Dysbiosis disrupts tight junctions and the mucus layer, increasing leakage of products such as LPS, activating inflammatory pathways. Some invasive bacteria further compromise barrier integrity, creating a vicious cycle (59). A more specific feature in IBD is the enrichment of adherent-invasive Escherichia coli (AIEC) (60). AIEC binds to abnormally overexpressed carcinoembryonic antigen-related cell adhesion molecule 6 on ileal epithelium via FimH adhesin, colonizing and invading the intestinal epithelium (61). Once engulfed by macrophages, AIEC can survive and replicate intracellularly, continually releasing high levels of TNF-α, leading to chronic intestinal inflammation (62).

In summary, both IgAN and IBD patients exhibit alterations in gut microbiota composition that correlate to varying degrees with disease activity and clinical severity. However, a substantial proportion of the evidence derives from cross-sectional or case-control studies, which cannot rule out the confounding effects of diet, geographical factors, or medication exposure. Moreover, inter-study differences in sequencing platforms may limit the comparability of results. Future prospective cohort studies and animal intervention experiments are warranted to further clarify the causal relationships involved.

### 4.2 The role of gut microbiota metabolites in the gut–kidney axis

Beyond compositional changes, dysbiosis is also accompanied by shifts in metabolic function, with microbial metabolites serving as key functional messengers in the gut–kidney axis (**Supplementary Figure 1**).

#### 4.2.1 Short−chain fatty acids

Short−chain fatty acids (SCFAs) are key immunomodulatory molecules in intestinal homeostasis. In IBD, butyrate promotes regulatory T cell (Treg) differentiation and function while inhibiting Th17−mediated inflammation via G protein–coupled receptor signaling and histone deacetylase inhibition (63). Studies have shown that fecal SCFA levels are significantly reduced in IgAN patients, correlating with clinical features and gut microbiota alterations (64). SCFAs may regulate inflammatory processes and exert renoprotective effects through analogous signaling pathways (65).

#### 4.2.2 Bile acids

Gut microbiota are involved in the biotransformation of bile acids, converting primary bile acids into secondary bile acids (66). Secondary bile acids can modulate intestinal immune homeostasis via nuclear receptors such as farnesoid X receptor (FXR) and G protein−coupled bile acid receptor 1 (GPBAR1) (67). Both receptors are highly expressed in the kidney. Activation of FXR attenuates inflammatory responses by inhibiting the NF−κB pathway, reduces mitochondrial reactive oxygen species production to alleviate oxidative stress, and suppresses the TGF−β/Smad pathway to reduce renal fibrosis. Activation of GPBAR1 participates in regulating lipid, cholesterol, and carbohydrate metabolism, as well as the expression of genes related to inflammation and fibrosis (68).

#### 4.2.3 Uremic toxins

When renal function declines, certain gut microbiota−derived metabolites accumulate in the body, including indoxyl sulfate (IS), p−cresyl sulfate (PCS), and trimethylamine N−oxide (TMAO). IS and PCS activate the EGFR signaling pathway, upregulate matrix metalloproteinase−2 and −9, and induce renal tubulointerstitial tissue remodeling (69). TMAO activates the NLRP3 inflammasome, stimulates macrophage activation and T−cell differentiation, and promotes the release of inflammatory cytokines such as TNF−α and IL−6. In parallel, it activates the NF−κB signaling pathway, Caspase−1, and the PERK/Akt/mTOR pathway, upregulating IL−1β and other pro−inflammatory and pro−fibrotic factors that promote renal fibrosis (70).

In IgAN and IBD, the emphasis may differ among reduced SCFAs, bile acid metabolic disturbances, and uremic toxin accumulation, yet all of them act as signaling carriers between the gut and the kidney, translating local mucosal immune abnormalities into systemic inflammation or tissue damage. In turn, after renal function declines, toxin accumulation further disrupts the intestinal barrier.

## 5 Treatment of IgAN and IBD

### 5.1 Current therapies for IgAN and IBD and restoration of gut microbiota

The 2025 Kidney Disease: Improving Global Outcomes (KDIGO) guideline explicitly proposes that the specific immune pathogenesis of IgAN and the secondary response following nephron loss should be treated as two independent but simultaneously managed therapeutic targets. Supportive care includes renin-angiotensin system inhibitor (RASi), sodium-glucose cotransporter-2 inhibitors, endothelin receptor antagonists, and mineralocorticoid receptor antagonists, while immunosuppressive therapy relies primarily on GC (71). Clinical studies have shown that GC treatment is associated with reduced serum total IgA, Gd-IgA1 levels, and proteinuria, suggesting a potential role for their anti-inflammatory and immunomodulatory effects (72, 73). In recent years, therapeutic strategies targeting gut microbiota and mucosal immunity have gained attention. Zhao et al. prospectively followed 77 IgAN patients to explore the association between gut microbiota changes and treatment response after six months of immunosuppressive therapy. They found that patients achieving clinical remission showed reversal of dysbiosis, with a significant decrease in *Escherichia-Shigella* levels (52). Another cross-sectional study also confirmed that the distinct microbial features in IgAN patients changed accordingly after immunosuppressive treatment (40). We propose that immunosuppressive therapy may correct local or systemic mucosal immune responses triggered by increased abundance of pathogenic bacteria. Once inflammation and dysbiosis are controlled, the clinical symptoms of patients may also be alleviated. However, GC and some immunosuppressants non-specifically suppress systemic immunity and have significant adverse effects with long-term use. Microecological interventions such as fecal microbiota transplantation (FMT) avoid these side effects while showing efficacy. In case reports, Zhao et al. first reported two patients with refractory IgAN who received intensive FMT; their 24h proteinuria markedly decreased, renal function remained stable, gut microbiota diversity was restored, *Proteobacteria* decreased, and *Prevotella* increased, achieving partial clinical remission (74). An exploratory clinical trial showed that FMT combined with RASi effectively reduced proteinuria and modulated plasma B-cell immune function (75). Animal experiments have also demonstrated that targeting these microbiota can attenuate IgAN kidney injury by repairing the intestinal barrier and suppressing B-cell overactivation (76). These findings suggest that restoring gut microecology may represent a potential approach to modulating mucosal immunity and mitigating glomerular inflammation, although further clinical studies are needed. The changes in microbiota before and after treatment are summarized in **Table 1**.

Treatment of IBD is stratified based on disease subtype and severity. 5-aminosalicylic acid (5-ASA) is first-line for mild-to-moderate UC, while moderate-to-severe disease requires GC, immunomodulators, and novel biologic agents (77). During treatment, microbiota restoration and immune reconstitution also occur. A longitudinal cohort study found that IBD patients who responded to infliximab (IFX) showed a significant increase in total bacterial load in feces and ileal mucosa, mainly reflected by an increase in butyrate-producing bacteria (78). The shift toward a healthy microbial phenotype facilitates metabolite recovery, which re-establishes the Th17/Treg balance and is closely associated with post-treatment mucosal healing and clinical improvement (79). Further studies showed that in CD patients responding to IFX, the proportion of peripheral blood Tregs and serum levels of TGF-β1 and IL-10 also significantly increased (80). At the mucosal level, IFX treatment reduced the number of CD68^+^ macrophages in the colonic lamina propria and markedly downregulated IL-17A expression; these changes were highly correlated with endoscopic response (81). These findings indicate that biologic agents such as IFX, while eliminating inflammation, also promote the recovery of beneficial microbiota and immune homeostasis. Moreover, case reports have shown that in patients with both IBD and IgAN, the severity of intestinal inflammation fluctuates synchronously with the degree of kidney injury. Effective control of intestinal symptoms may be accompanied by improvement in renal manifestations (8).

### 5.2 Budesonide enteric-coated capsules: etiological treatment targeting the gut origin of IgAN

**Figure 3:**
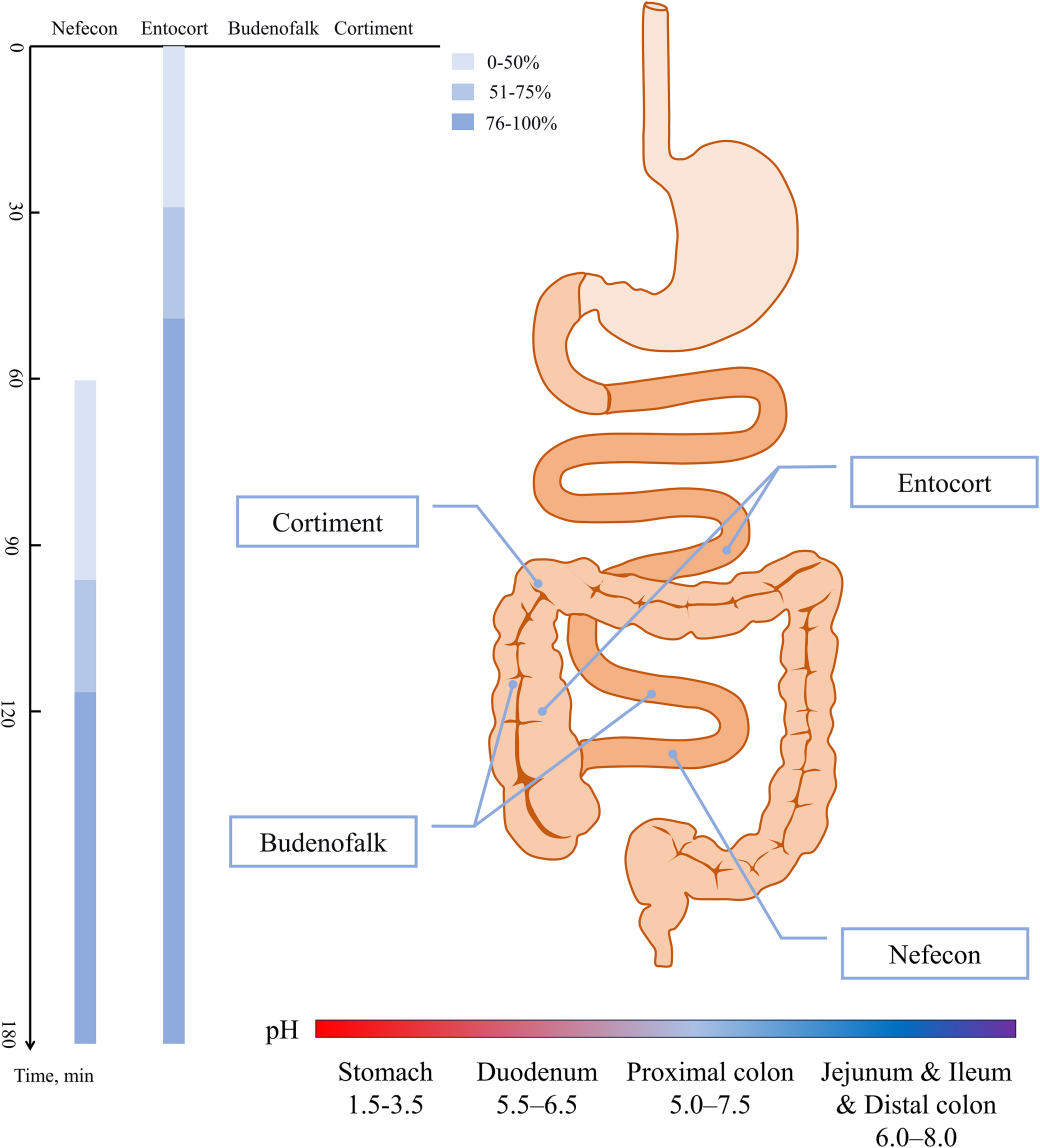
Release sites of oral budesonide formulations in the intestine. The release of four marketed oral budesonide formulations—Nefecon, Entocort, Budenofalk, and Cortiment—depends on the pH of different intestinal segments. *In vitro* studies have simulated the mean release rate of several formulations within three hours after intestinal entry (84).

Given the close relationship between gut microbiota and mucosal immunity, intervention strategies targeting local intestinal immunity have shown significant efficacy in treating IgAN. The KDIGO 2025 guideline clearly states that budesonide enteric capsules are currently the only drug proven to reduce pathogenic IgA and IgA immune complexes, and are recommended for IgAN patients at risk of progression (71). The global phase III NefIgArd study demonstrated that 9 months of treatment with budesonide enteric capsules significantly slowed eGFR decline and persistently reduced proteinuria (82). Data from the Chinese subgroup yielded results consistent with the global study population (83). Although the currently marketed oral budesonide formulations (Nefecon, Entocort, Budenofalk, and Cortiment) share the same active ingredient, they differ in dosage form and release mechanism (**Table 3**). The differences in drug release mechanisms primarily stem from the pH value that triggers release, which determines whether the drug can act at the target site. The action sites of the four oral budesonide formulations partially overlap: Entocort releases in the ileum and ascending colon at pH > 5.5; Budenofalk and Cortiment require a higher release pH and mainly target the colon (**Figure 3**). Nefecon features a three-layer coating structure that effectively prolongs drug action time, bypasses the stomach and upper small intestine, and precisely delivers budesonide to the terminal ileum rich in Peyer’s patches, thereby inhibiting aberrant B-cell activation and reducing Gd-IgA1 production and IgA- immune complexes formation at the source (84, 85). Peyer’s patches are not only sites of B-cell activation but also key locations for intestinal T-cell immune regulation. In IgAN patients, the proportion of Th17 cells in peripheral blood is elevated, while that of Tregs is reduced, and this imbalance is closely associated with proteinuria levels and declining renal function (86). Cytokines related to Th2, Th17, and Tfh cells can promote the synthesis of Gd-IgA1 (87). Therefore, targeting Peyer’s patches to modulate local mucosal immunity may not only affect aberrant B-cell activation but also help improve local T-cell immune homeostasis. However, direct evidence for a modulatory effect of budesonide on the Treg/Th17 balance is currently lacking, and this hypothesis warrants further investigation. *In vitro* dissolution profiles show that Nefecon is not released within 2 hours in simulated gastric fluid; after transfer to intestinal fluid, it disintegrates in about 1 hour and most of the drug is released within the following 2 hours. The other three formulations exhibit sustained or extremely slow release (84). Thus, Nefecon’s release behavior differs from that of conventional formulations, and its core targeted-release technology provides a pharmacokinetic basis for selectively modulating gut mucosal immunity.

**Table 3:** Summary of four oral budesonide formulations.

Item	Nefecon	Other oral budesonide formulations		
		Entocort	Budenofalk	Cortiment
Indication	IgAN	Crohn’s disease Microscopic colitis	Crohn’s disease Autoimmune hepatitis Microscopic colitis	Ulcerative colitis Microscopic colitis
Dosage form	Enteric-coated capsule	Enteric-coated capsule containing coated pellets	Enteric-coated capsule with pH-dependent coated pellets	Tablet
Release pH	> 6.5	> 5.5	> 6.4	≥ 7.0
Release site	Terminal ileum	Ileum and ascending colon	Ileum and ascending colon	Entire colon
Core technology	Targeted-Release	pH-dependent and time-dependent dual controlled release	pH-dependent release	Multi matrix system technology
Pharmacokinetics	Delayed release and prolonged Tmax Higher peak plasma concentration	Rapid release and short Tmax Lower peak plasma concentration	Intermediate between Entocort and Nefecon	Extremely delayed release and markedly prolonged Tmax Stable plasma concentration
Dosage and duration	Dosage: 16mg once daily Treatment duration: 9 months	Dosage: 9mg once daily Treatment duration: 8 weeks		

Adapted with modifications from Barratt J, Kristensen J, Pedersen C, Jerling M. Insights on Nefecon®, a targetedrelease formulation of budesonide and its selective immunomodulatory effects in patients with IgA nephropathy. Drug Des Devel Ther (84).

Budesonide enteric capsules target gut mucosal immunity, yet mucosal responses are not confined to the gut. The frequent occurrence of macroscopic hematuria following upper respiratory infections in IgAN patients highlights the involvement of the tonsils. Thus, tonsil-directed therapy also warrants further investigation.

### 5.3 Tonsillectomy: targeting tonsillar mucosal immunity

Dysbiosis activates GALT via relevant signaling pathways, constituting a gut-derived mechanism in IgAN pathogenesis. The tonsils, as core organs of nasopharyngeal-associated lymphoid tissue, can similarly trigger a pathogenic IgA1 response under recurrent infection or dysbiosis, establishing a respiratory-derived pathway. B-cell activating factor (BAFF) and a proliferation−inducing ligand (APRIL) are key mediators in this pathway. Both bind to TACI, BCMA, and BAFF-R receptors on B cells, driving B-cell survival, class switching, and plasma cell differentiation (88). A meta-analysis revealed that serum levels of both molecules are significantly elevated in IgAN patients and correlate with disease severity (89). In the tonsils, recurrent infection or microbial stimulation induces APRIL production in tonsillar B cells via TLR9, while activated dendritic cells and follicular dendritic cells within germinal centers secrete BAFF and APRIL, thereby promoting B-cell survival and IgA class switching, leading to excessive Gd−IgA1 production (90–92). Activated submucosal innate immune cells release large amounts of pro-inflammatory cytokines, exerting a “trigger effect” that initiates an inflammatory storm and exacerbates kidney injury (**Figure 2**). This explains why IgAN patients frequently develop macroscopic hematuria shortly after an upper respiratory infection in clinical practice. Moreover, leukemia inhibitory factor can also enhance Gd-IgA1 production by tonsil-derived IgA1-secreting cells via the JAK2/STAT1 signaling pathway (90, 91, 93). Tonsillectomy combined with immunosuppressive therapy has been considered a treatment option for IgAN, particularly in Japan. A meta-analysis of 858 patients showed that tonsillectomy plus GC significantly increased the remission rate, with sustained benefits during long-term follow-up (94). Compared with immunosuppression alone, tonsillectomy combined with immunosuppressive therapy significantly improved and accelerated clinical remission, reduced relapse rates, and delayed disease progression (95). However, most of the above evidence originates from retrospective studies conducted in Japan, lacks validation at the level of randomized controlled trials, and may be subject to the influence of geographical differences, patient selection bias, and applicability in different healthcare settings. Therefore, it remains unclear whether the results can be generalized to patients in other regions. In summary, post-infection tonsillar responses drive Gd-IgA1 production and renal deposition through their unique mucosal immune environment, ultimately leading to IgAN.

## 6 Unresolved issues and future perspectives

Why do similar genetic backgrounds and dysbiosis lead to different diseases? Possible explanations include differences in antigen exposure or sites of mucosal immune initiation. A large Japanese cohort reported higher IgAN risk in IBD patients (96), yet not all IBD patients develop IgAN. We propose two possible explanations. First, the extent of pathogenic IgA production may differ: intestinal B cells in some IBD patients may be more prone to producing Gd-IgA1, whereas others predominantly exhibit Th17/Treg-mediated inflammation. Second, the ease with which IgA leaks into the circulation after intestinal barrier disruption may vary: the severity of barrier injury, repair capacity, and the efficiency of local immune complexes clearance could affect systemic exposure to pathogenic IgA. The above are preliminary hypotheses, and prospective cohort studies directly comparing IBD patients with and without concurrent IgAN remain lacking.

Both the gut and tonsils are considered sources of Gd-IgA1, yet the quantitative contribution of each remains unclear. Recent multi-omics studies suggest that the terminal ileum may be a major source of Gd-IgA1 (97), but direct evidence regarding the relative contribution of the gut versus the tonsils is still needed. Accurate quantification of the contribution from different mucosal sites to pathogenic IgA production would help guide mucosa-targeted therapeutic strategies.

As the evidence presented above suggests, gut microbiota dysbiosis is present in IgAN, but whether it represents a driving factor of the disease or a secondary manifestation of inflammation remains unresolved. Moreover, given that most available evidence is derived from cross-sectional studies, causality cannot be established. Nevertheless, diagnostic models based on gut microbiota features have shown some potential (98, 99). However, due to the multiple factors influencing microbiota composition, it is currently insufficient as a reliable non-invasive biomarker, and its value as an adjunctive risk stratification tool warrants further exploration.

## 7 Conclusion

IgAN is a chronic disease requiring lifelong management. If dysbiosis and related immune imbalance persist, the disease may progress continuously. IgAN and IBD share some genetic loci and both exhibit dysbiosis. Therefore, future studies should further explore the therapeutic potential of restoring the imbalanced gut microecology and re-establishing immune homeostasis, in addition to controlling inflammation. Moreover, the role of tonsillar and other respiratory mucosal immunity in pathogenesis should not be overlooked.
