# Supplementary material for: Gut microbiota dysbiosis and immune responses: insights from IgA nephropathy and inflammatory bowel disease
Source: Front Immunol. 2026 Jul 29;17:1889646. doi: 10.3389/fimmu.2026.1889646 (PMC13462605; doi:10.3389/fimmu.2026.1889646)
Supplement: Supplementary file 1 [file Supplementaryfile1.docx]

Supplementary Material

# 1 Supplementary Figure

**Figure S1.** Gut microbiota metabolites and the gut–kidney axis. Gut microbiota decompose dietary substrates to produce short-chain fatty acids, secondary bile acids, and uremic toxins. These metabolites are released into the circulation and regulate renal inflammation and fibrosis through corresponding receptors or inflammatory pathways. Impaired renal function leads to toxin accumulation, which damages the intestinal barrier and establishes a gut–kidney vicious cycle. SCFAs, short-chain fatty acids; BAs, bile acids; IS, indoxyl sulfate; PCS, p-cresyl sulfate; TMAO, trimethylamine N-oxide.
